# Study protocol for the Multimodal Approach to Preventing Suicide in Schools (MAPSS) project: a regionally based randomised trial of an integrated response to suicide risk among secondary school students

**DOI:** 10.1186/s13063-022-06072-8

**Published:** 2022-03-02

**Authors:** Sadhbh J. Byrne, Eleanor Bailey, Michelle Lamblin, Samuel McKay, Jane Pirkis, Cathrine Mihalopoulos, Matthew J. Spittal, Simon Rice, Sarah Hetrick, Matthew Hamilton, Hok Pan Yuen, Yong Yi Lee, Alexandra Boland, Jo Robinson

**Affiliations:** 1 Orygen, Parkville, Australia; 2 Centre for Youth Mental Health, The University of Melbourne, Melbourne, Australia; 3 Centre for Mental Health, Melbourne School of Population and Global Health, The University of Melbourne, Melbourne, Australia; 4 Deakin Health Economics, Institute for Health Transformation, School for Health and Social Development, Faculty of Health, Deakin University, Geelong, Australia; 5 Department of Psychological Medicine, The University of Auckland, Auckland, New Zealand; 6 School of Public Health, The University of Queensland, Brisbane, Australia; 7 Policy and Epidemiology Group, Queensland Centre for Mental Health Research, Brisbane, Australia

**Keywords:** Suicide prevention, Schools, Psychoeducation, Screening, iCBT

## Abstract

**Background:**

Suicide is the leading cause of death among young Australians, accounting for one-third of all deaths in those under 25. Schools are a logical setting for youth suicide prevention activities, with universal, selective and indicated approaches all demonstrating efficacy. Given that international best practice recommends suicide prevention programmes combine these approaches, and that to date this has not been done in school settings, this study aims to evaluate a suicide prevention programme incorporating universal, selective and indicated components in schools.

**Methods:**

This study is a trial of a multimodal suicide prevention programme for young people. The programme involves delivering universal psychoeducation (safeTALK) to all students, screening them for suicide risk, and delivering internet-based Cognitive Behavioural Therapy (Reframe IT) to those students identified as being at high risk for suicide. The programme will be trialled in secondary schools in Melbourne, Australia, and target year 10 students (15 and 16 year-olds). safeTALK and screening will be evaluated using a single group pre-test/post-test case series, and Reframe IT will be evaluated in a Randomised Controlled Trial. The primary outcome is change in suicidal ideation; other outcomes include help-seeking behaviour and intentions, and suicide knowledge and stigma. The programme’s cost-effectiveness will also be evaluated.

**Discussion:**

This study is the first to evaluate a suicide prevention programme comprising universal, selective and indicated components in Australian schools. If the programme is found to be efficacious and cost-effective, it could be more widely disseminated in schools and may ultimately lead to reduced rates of suicide and suicidal behaviour in school students across the region.

**Supplementary Information:**

The online version contains supplementary material available at 10.1186/s13063-022-06072-8.

## Administrative information

**Table:** 

**Title**	Study protocol for the Multimodal Approach to Preventing Suicide in Schools (MAPSS) project: A regionally-based trial of an integrated response to suicide risk among secondary school students.
**Trial registration**	Australian New Zealand Clinical Trial Registry, ACTRN12621000279820 12.03.2021 https://www.anzctr.org.au/Trial/Registration/TrialReview.aspx?id=381921&isClinicalTrial=False
**Protocol version**	24^Th^ November 2021, version 2.0
**Funding**	This research is funded by the National Health and Medical Research Council (NHRMC), Australia (GNT1153051). [See Additional file 1 for copy of the original funding documentation]
**Author details**	^1^Orygen, Parville, Australia ^2^Centre for Youth Mental Health, The University of Melbourne, Melbourne, Australia ^3^Centre for Mental Health, Melbourne School of Population and Global Health, The University of Melbourne, Melbourne, Australia ^4^Deakin University, Geelong, Deakin Health Economics, Institute for Health Transformation, School for Health and Social Development, Faculty of Health, Australia ^5^Department of Psychological Medicine, The University of Auckland, Auckland, New Zealand ^6^School of Public Health, The University of Queensland, Brisbane, Australia ^7^Policy and Epidemiology Group, Queensland Centre for Mental Health Research, Brisbane, Australia
**Name and contact information for the trial sponsor**	Orygen, 35 Poplar Road, Melbourne, Australia + 61 3 9342 2800 research@orygen.org.au
**Role of sponsor and funder**	This is an investigator-initiated research project which has been funded by non-commercial sources. The role of the funder (National Health and Medical Research Council) and Sponsor (Orygen) will not impact on decisions regarding the outcome of this research or how it will be published. Orygen (as Sponsor) has taken an active role in the monitoring of the study as both Orygen and the funder require the studies to be conducted in accordance with local regulatory guidelines and industry best practice. However, the research and its outcomes are independent of both the Sponsor and the Funder.

## Background

Suicide is the leading cause of death in young Australians, accounting for more than one-third of all deaths in those under 25 [1]. Suicidal ideation and behaviour, including non-fatal suicide attempts and self-harm, are more common and are associated with a host of negative outcomes including risk of future suicidal behaviour and suicide [2, 3]. The impact of suicide on a young person’s family, friends, and wider community can be devastating and may include increased risk of suicidal behaviour and suicide [4]. There is therefore an urgent need to develop and test acceptable and accessible approaches to preventing suicide and suicidal behaviours in this population.

Schools are an appropriate and convenient setting for the delivery of youth suicide prevention programmes [5] and have been identified as important locations for suicide prevention activities by national and international suicide prevention strategies [6, 7]. Although school wellbeing staff may be well-placed to provide support to students at risk of suicide, young people are often reluctant to seek help from professionals and instead turn to their friends for support [8]. School-based prevention efforts must therefore not only target school staff, but also fellow students. Historically, there has been a reluctance to deliver suicide prevention efforts to students, due to concerns about the potentially iatrogenic impact of asking or talking about suicide [9, 10]. However, increasing evidence suggests that it is safe to do [11–13]. Moreover, a recent systematic review and meta-analysis [14] revealed that school-based suicide prevention programmes should combine universal, selective, and indicated approaches in order to be most effective. Each approach as it relates to school settings is reviewed briefly below.

### Universal approaches

Universal approaches target whole populations, regardless of risk [15]. In school settings, these typically involve psychoeducation programmes, which aim to increase suicide literacy and reduce stigma, encourage help-seeking, and equip participants with skills to support peers [16]. A number of psychoeducation suicide prevention programmes have been tested internationally and appear to increase knowledge, attitudes, and help-seeking intentions; some have also been associated with reductions in suicidal ideation and behaviour [17–21]. One example is LivingWorks safeTALK, a 3.5-h workshop suitable for delivery to anyone over the age of 15. The programme has been positively evaluated in adult populations [22–25]. In 2015, our team conducted a pilot evaluation of safeTALK in secondary schools in Alice Springs, Australia, with 129 students (mean age = 16.7 years). The intervention was associated with increased knowledge about suicide, confidence to intervene with a suicidal person, and willingness to seek help and help others. No iatrogenic effects were reported [26].

### Selective approaches

Selective approaches aim to prevent the onset of suicidal thoughts or behaviour in populations at elevated risk of suicide [27]. In school settings, these include training gatekeepers, such as teachers, to recognise and intervene with students at risk, and screening students for suicide risk followed by referral to relevant support. Screening can facilitate the identification of at-risk students, including those who have not previously sought help, and has not been associated with iatrogenic effects [28–30]. As part of the evaluation of safeTALK described above, screening questions were embedded into the questionnaires to identify students potentially at risk of suicide. Almost half of the participants screened positive, many of whom had not previously sought help, and were referred for further support [26].

### Indicated approaches

Indicated approaches target those individuals already displaying suicidal thoughts and/or behaviours and include face-to-face psychotherapy, pharmacotherapy, and more recently, online programmes. A range of online programmes targeting suicidal thoughts have been developed and evaluated, with promising results [31, 32]. The “Reframe IT” programme was developed by our team in 2011. Reframe IT is an online cognitive behavioural therapy (CBT) programme specifically designed for young people presenting to the school counsellor with suicide risk. Reframe IT was piloted in two phases. First, a pre-test post-test case series study found it to be associated with reduced suicidal ideation, depression, and hopelessness [33]. Second, in a pilot randomised controlled trial, the intervention group showed larger (non-significant) mean improvements in suicidal ideation, depression, and hopelessness, when compared to controls [34]. Participants in both studies provided positive feedback about Reframe IT, and it was not associated with any iatrogenic effects [33–35].

### Integrated approaches to suicide prevention

According to international best practice, suicide prevention programmes should incorporate universal, selective, and indicated approaches [15]. Such approaches have shown promise in both community and school settings, although the majority of those in school settings combine only two levels of prevention, such as universal (e.g. psychoeducation) and selective (e.g. screening) components [14]. There is therefore a need to develop, implement, and evaluate an approach combining universal, selective, and indicated approaches in school settings. To enable ongoing implementation and dissemination of such a programme, evidence must support not only its efficacy but also its cost-effectiveness. To date, no studies (Australian or otherwise) have applied rigorous economic methodology to evaluate short- and longer-term comparative cost-effectiveness of an intervention comprising universal, selective, and indicated elements.

### Aims and hypotheses

This project aims to evaluate the effectiveness of an integrated programme comprising universal, selective, and indicated components in reducing suicide risk, improving risk recognition, and increasing health service use among young people across North West Melbourne. Secondary aims are to evaluate the programme’s safety and cost-effectiveness. The programme involves the delivery and evaluation of universal educational workshops (safeTALK) to school students plus screening students for suicide risk (universal and selective approaches; Component 1), and the evaluation of online CBT (Reframe IT) delivered to students identified as being at risk (indicated approach; Component 2). Several hypotheses have been generated relating to each of the project components as well as the project as a whole.

It is hypothesised that completion of the safeTALK workshop will be associated with increased intentions to intervene with a suicidal person, higher levels of suicide literacy, higher levels of perceived behavioural control regarding intervening with a suicidal person, and reduced stigma towards suicide and suicidal behaviour.

It is hypothesised that the screening will lead to the identification of students at risk of suicide-related behaviour who had not previously sought help.

It is hypothesised that, compared to the control group, students in the Reframe IT intervention group will demonstrate reduced suicidal ideation, reduced symptoms of depression, reduced hopelessness, and improved problem-solving.

It is hypothesised that the project as a whole will lead to increased health-service use; increased help-seeking from informal sources; and improved attitudes towards help-seeking.

Finally, it is hypothesised that the programme will be cost-effective and have no iatrogenic effects.

## Methods

### Study design

The present study will adopt two methodological approaches: Component 1 will employ a single-group, pre-test/post-test case series; Component 2 is a parallel superiority randomised controlled design, where the intervention condition (Reframe IT plus treatment as usual; TAU) is compared to TAU. Component 2 will be conducted and reported according to the Consolidated Standards of Reporting Trials (CONSORT) statement [36].

The study was designed to be conducted over a 5-year period (2019–2023), with a 3-year intervention phase. There has, however, been a 1-year suspension due to the COVID-19 pandemic during 2020, meaning that the study will continue into 2024.

The study is registered with the Australian New Zealand Clinical Trials Registry (ID: ACTRN12621000770864). This is the primary registry of the WHO ICTRP network. It includes all items from the WHO Trial Registration data set, available at https://www.anzctr.org.au/Trial/Registration/TrialReview.aspx?id=381921&isClinicalTrial=False. It received ethical approval from the University of Melbourne Human Research Ethics Committee (HREC; ID 1852317) and the Department of Education Victoria Research Ethics Committee (ID 2019_003951) [see Additional files 2 and 3].

This protocol is reported in line with the Standard Protocol Items: Recommendations for Interventional Trials (SPIRIT) 2013 statement [37, 38] [see Additional file 4 for checklist].

### Setting

This study is led by researchers at Orygen in Melbourne, Australia. Orygen is a youth mental health organisation with expertise in research, policy, education, and innovations in care. Since 1984, Orygen has been working side-by-side with young people to reduce the impact that mental ill-health has on young people, their families and society. Orygen services the north and west metropolitan areas of Melbourne, which, with a population of approximately 1.67 million, is one of the fastest growth corridors in Australia [39, 40]. It includes 31% of the state’s total population [40] and 11 of Melbourne’s 14 most disadvantaged municipalities, and reports high levels of mental illness [39, 40].

Orygen research staff are part of the Centre for Youth Mental Health, University of Melbourne. This project is a partnership with Lifeline Australia and the Department of Education and Training Victoria. Other collaborators are the University of Melbourne School of Population and Global Health, Deakin University, and the University of Auckland.

The catchment area for the current study aligns with the four geographical areas that comprise north and west metropolitan Melbourne, according to the regional model operated by the Department of Education and Training Victoria and the Department of Health and Human Services Victoria as well as Orygen’s clinical catchment area [41, 42]. Mainstream secondary schools that are located in the study catchment area and have year 10 students enrolled will be eligible as study sites; at the time of publication, 141 schools meet these criteria. A list of participating schools will be maintained.

### Participants

Participants will primarily be consenting year 10 students (i.e. aged 15–16) from secondary schools in north and west Melbourne. Year 11 students will also be included if the school perceives a strong need for delivery of the programme to this year level, or if we struggle to meet the required sample size (see section “Sample size and power calculation” of this paper). Exclusion criteria are intellectual disability and inability to converse in or read English. The additional inclusion criterion for participation in Component 2 (Reframe IT randomised controlled trial) is past 4-week suicidal ideation, as indicated by either a score of 21 or higher on the Suicidal Ideation Attributes Scale (SIDAS), or a score of 1–20 confirmed by a screening interview with a researcher.

#### Plans to promote participant retention and complete follow-up

Each participating school will be invited to nominate key staff members to complete Applied Suicide Intervention Skills Training (ASIST), delivered free-of-charge by Lifeline facilitators, to increase staff capacity to enact safety protocols. We hope this will also act as an incentive for schools to participate. The study team will take all reasonable steps to minimise any potential burden on school staff. Periodic communications will be provided to school leadership via newsletters and presentations to update on the progress of the study, and to acknowledge their support. Upon completion of the study, schools will also be provided with an anonymised aggregate report of the questionnaire data collected.

### Discontinuation and withdrawal

Participants who are withdrawn or discontinued from the study will not be replaced.

#### Voluntary discontinuation

Participants are free to discontinue their participation at any time. Participants who discontinue from the study will always be asked about the reason(s) for their discontinuation and about the presence of any adverse events. All adverse events will be followed up until resolution or the participant is lost to follow-up.

#### Participant discontinuation criteria

A participant will be considered discontinued from Component 1 if they do not attend the safeTALK workshop for any reason (including if they leave the workshop in the first 30 min). Participants who arrive more than 30 min late to the safeTALK workshop will miss important safety and wellbeing information, and will not be permitted to continue; they will also be considered discontinued from Component 1. Given that some students in Component 1 will also be participating in Component 2, all students regardless of their safeTALK completion will be asked to complete the subsequent questionnaires.

If a participant leaves the workshop early, a member of the study team will record how much of the workshop they completed and use this to determine whether the participant is considered “withdrawn”.

A participant will be discontinued from the study if they miss the T1 assessment. If a student consents but misses the baseline assessment, they may complete the baseline any time prior to T2, after which they will be discontinued.

A participant allocated to the intervention group in component 2 will be discontinued if:

1. Use of the intervention interferes with appropriate clinical management of risk of harm to self or others (as judged by the school wellbeing staff and/or senior researchers);
2. Serious adverse events (SAEs; defined in section “Definitions of adverse events and serious adverse events”) occur that could be associated with the Reframe IT intervention;
3. The participant or their parent or guardian indicates they no longer wish to use the Reframe IT intervention.

#### Participant withdrawal criteria

A participant will be considered “withdrawn” from the study in cases where all involvement is ceased.

A participant will be withdrawn if:

1. Participation interferes with appropriate clinical management of risk of harm to self or others (as judged by the school wellbeing staff and/or senior researchers, and in consultation with treating clinicians if appropriate);
2. SAEs occur that could be associated with any intervention included in this study;
3. The participant or their parent or guardian indicates they no longer wish to participate;
4. The participant leaves the school.

#### Study discontinuation criteria

The study may be terminated by the Sponsor (Orygen) with or without cause.

Discontinuation will be considered in the event that a suicide attempt or death occurs, and it is *reasonably possible* that the attempt or death was caused by any of the interventional components (safeTALK, screening or Reframe IT) of the study. See section “Assessment and documentation of AEs”. for details on determination of causality.

### Recruitment

#### Recruitment of schools

Pending approval from their regulating authorities, all mainstream secondary schools located in the study catchment area with year 10 students enrolled will be invited to participate via letters of invitation and direct contact from the research team.

#### Recruitment of students

##### Component 1

All students in year 10 (or year 11, where applicable) at participating schools will be invited to participate. Students will be informed about the study in a number of ways, including announcements at assembly, letters to parents, parent and student information evenings, and participant information sheets. They will receive a full explanation of the aims of the study, the potential discomfort, risks, and benefits of taking part. It will be explained that the study is for research purposes and may provide benefit to the individual and that they can withdraw from the study at any time. Participants will also be asked to provide additional consent for the use of their data for future research. The use of their data for future research is optional, so participants may still take part in the current study without consenting to their data being used in future research projects. Participants will be notified that any future research projects wanting to use their data will have to be reviewed and approved by a recognised HREC. Written consent will be required from students, plus parents or legal guardians, prior to participation at T1, through completion of a study consent form. [See Additional file 5 for a sample Participant Information and Consent Form (PICF)]. The participant will be given a copy of the signed PICF to retain.

##### Component 2

Eligible students will be invited to participate either by a school counsellor or a member of the research team. Students and their parents or guardians will be required to sign a separate PICF, within 2 weeks of the baseline assessment.

### Procedure

Participants will be assessed at four time-points: baseline (T1), 2 weeks post-baseline (T2), 12 weeks post-baseline (T3), and 24 weeks post-baseline (T4). The T2 assessment will be completed immediately following participation in the safeTALK workshops, and T3 at the end of the Reframe IT intervention period (see Table 1).

**Table 1:** Schedule of assessments and interventions

Time point		Study period				
	− T1	T1	T2		T3	T4
		Baseline Week 1	Week 3	Weeks 4–12	Week 12	Week 24
**Enrolment:**						
Informed Consent—safeTALK	X					
Inclusion/Exclusion Criteria—Reframe IT		X				
Informed Consent—Reframe IT			X*			
**Intervention administered:**		**Screening**	**safeTALK**	**Reframe IT** (one module per week)		
**Assessments:**						
Demographics		X				
Purpose-designed 3-item questionnaire assessing exposure to suicide and suicide prevention training		X				
Suicidal Ideation Attributes Scale (SIDAS)		X	X		X	X
Single item measuring current suicidal ideation		X	X		X	X
Youth Risk Behaviour Survey (YRBS)—suicide items		X	X		X	X
Patient Health Questionnaire-9 (PHQ-9)		X	X		X	X
Distress Questionnaire-5 (DQ-5)		X				
Hopelessness Scale for Children (HSC)		X	X		X	X
Negative Problem Orientation Questionnaire (NPOQ)		X	X		X	X
Resource Use Questionnaire (RUQ), adapted from the Young Mind Matters Service Use questionnaire (BRIEF VERSION)		X				
Resource Use Questionnaire (RUQ), adapted from the Young Mind Matters Service Use questionnaire (FULL VERSION)			X		X	X
Purpose-designed questionnaire asking about previous help-seeking behaviour and future help-seeking intentions from family, friends and other “informal” sources of help		X	X		X	X
4-item measure of attitudes towards help-seeking from adults at school, derived from the previous US Sources of Strength (SOS) trial and currently being used in the Australian SOS trials		X	X		X	X
4-item measure assessing attitudes towards overcoming secrecy barriers, derived from the previous US Sources of Strength (SOS) trial and currently being used in the Australian SOS trials		X	X		X	X
Willingness to Intervene Against Suicide Questionnaire (WIS)		X	X		X	X
Stigma of Suicide Scale (SOSS)—short form		X	X		X	X
Literacy of Suicide Scale (LOSS)—short form		X	X		X	X
Child Health Utilities Index 9 Dimension (CHU9D)		X	X		X	X
Purpose-designed 7-item questionnaire assessing acceptability of safeTALK training			X			
Purpose-designed 11-item questionnaire assessing acceptability of the Reframe IT intervention (or of not receiving it)					X	
Two items from the Patient Health Questionnaire-4 (PHQ-4), measuring anxiety		X				
Purpose-designed 11-item scale measuring experimentally induced distress, adapted from Yeater, Miller, Rinehart and Nason (2012)						X

*Note. Informed consent for Reframe IT participation and evaluation will be collected from eligible participants between Times 1 and 2, prior to randomisation and commencement of the Reframe IT intervention

Assessments will take the form of self-report questionnaires, and where possible will be collected using a computer or tablet employing REDCap (Research Electronic Data Capture), a secure, web-based data capture application hosted at The University of Melbourne [43, 44]. Where questionnaires are completed on paper, these will be later entered into REDCap by a member of the research team. Assessments will be completed at school in class groups, facilitated by a researcher, and supervised by a school staff member.

The REDCap system will automatically flag any participants who score between 1 and 20 on the SIDAS, as 1 is the established cut-off score indicating any level of suicidal ideation in the past month [45]. In the case that participants are required to complete paper versions of the questionnaire, they will be asked to include their full name on a separate detachable cover sheet. After the survey is completed, research staff will manually check their scores on the SIDAS instrument.

Within five school days of the baseline assessment, the research team will contact students who score between 1 and 20 on the SIDAS and conduct a brief semi-structured interview to determine eligibility for Component 2. Eligible students will be invited to participate and those who are interested will be given a consent form to be signed. They will then be randomised to either the intervention (Reframe IT) or control group (see below). A member of the research team will inform the relevant school staff member of the outcome of the randomisation.

Two weeks after the baseline assessment, safeTALK workshops will be delivered to all participating students. Immediately following the workshop, students will complete the T2 questionnaire. Students randomised to receive the Reframe IT intervention will complete the eight modules in the 10 weeks between T2 and T3 (i.e. approximately one module per week plus two additional weeks to allow for disruptions such as school holidays). Both T3 and T4 assessments will occur during class time; T4 will occur 12 weeks after T3. Researchers will be present at T3 and T4 assessment points if deemed appropriate by the research team and/or if requested by the school (e.g. they perceive a need for additional administrative support). No biological samples will be collected.

### Interventions

#### safeTALK

safeTALK, developed by LivingWorks Education [46], is a suicide alertness training workshop, suitable for anyone over the age of 15. It comprises a single 3.5-h face-to-face workshop, designed to help participants understand suicide warning signs in themselves and others, gain knowledge about appropriate sources of help for suicidal feelings, apply basic “TALK” steps (Tell, Ask, Listen, and KeepSafe), and feel better able to connect a person at risk of suicide with appropriate help. safeTALK workshops will be delivered by trained Lifeline Australia facilitators to classroom-sized groups of students (maximum of 30 students per session). A researcher will attend each workshop.

#### Screening for risk

The screening will take the form of self-report measures embedded into the questionnaires at each time point. Students who report suicidal ideation within the past 4 weeks (SIDAS score of 1 or higher) or any level of current suicidal ideation (single multiple-choice item) will be contacted by a member of the school wellbeing team or the research team, who will assess risk and refer them to appropriate support.

#### Reframe IT

The Reframe IT intervention has been described elsewhere [33–35]. In brief, it comprises eight 20-min self-guided CBT modules. It follows the stories of two young people who make video diaries about their day-to-day life and their experience of feeling suicidal. An adult “host” character guides the user through the module and activities. Each module contains two “activities” based on standard CBT exercises. Users progress sequentially through the content, with modules automatically unlocked when the preceding module is complete. There is also a message board through which the participant can communicate with a moderator, a mood diary function, and a series of factsheets and information on local and national helplines and services.

#### Control intervention

Participants randomised to the control group will receive TAU (e.g. from the school counsellor or external mental health services). The specific nature of TAU will be determined by the wellbeing team at each school. Although this may result in variability in treatment type between sites, the use of TAU as a comparator will facilitate an evaluation of whether receipt of the Reframe IT intervention is associated with improvement over current practice [47]. The wellbeing team at each school will be asked to record what TAU comprised for each student.

#### Strategies to improve adherence to intervention protocols

The safeTALK intervention will be delivered by qualified Lifeline facilitators, following a standardised format. The study team will provide on-boarding training to school wellbeing staff regarding the use of the Reframe IT online platform, and additional training sessions will be offered as required. Each participating school will be provided with a study manual.

#### Procedures for monitoring compliance with the intervention

To monitor compliance with safeTALK, a question will be included in the T2 questionnaire asking if participants were present for the entire safeTALK workshop. If participants respond “no”, they will be asked to indicate how much of the session they were present for. A researcher will be present for the delivery of safeTALK and will keep a record of any students who leave the session prematurely, including the amount of time and number of workshop modules the participant was present for.

Compliance with the screening intervention will be determined by assessing whether or not participants complete the suicide risk questions.

Participants’ use of Reframe IT (including a number of log-ins, number of modules completed, and responses to activities) will be automatically recorded by the system. At the conclusion of the study, usage statistics will be evaluated.

#### Concomitant medication and therapies

The nature of the study population is such that some participants may already be taking prescribed medications. There are no restrictions to participation based on concomitant medication or therapies.

### Outcomes and measures

#### Primary outcome

The primary outcome for this study is change in past 4-week suicidal ideation at T3 and T4 when compared to T2, assessed via the SIDAS [45]. The SIDAS is designed to screen individuals in the community for presence of suicidal thoughts and assess the severity of these thoughts. It comprises five items, each targeting an attribute of suicidal thoughts: frequency, controllability, closeness to attempt, level of distress associated with the thoughts and impact on daily functioning. Responses are measured on a 10-point scale. Total SIDAS scores are calculated as the sum of the five items, with controllability reverse scored (10 = 0, 9 = 1,…, 0 = 10), with total scale scores ranging from 0 to 50. Items are coded so that a higher total score reflects more severe suicidal thoughts.

#### Secondary outcomes

Change in symptoms of depression at T3 and T4 when compared to T2 will be assessed using the Patient Health Questionnaire—9 item version (PHQ-9) [48]. Participants will be asked to indicate how often they have been bothered by nine problems over the past 2 weeks. Each item is rated on a 4-point Likert scale ranging from 0 (“not at all”) to 3 (“nearly every day”). Scores are summed such that the potential range is 0–27, with higher scores indicative of greater distress.

Changes in hopelessness at T3 and T4 when compared to T2 will be assessed using the Hopelessness Scale for Children (HSC) [49], a 17-item measure of the occurrence of cognitions related to hopelessness within the past week. Participants rate each item as true or untrue of them; items 1, 3, 4, 5, 6, 7, 11, and 16 are scored as reflecting hopelessness if rated “false” and items 2, 8, 9, 10, 12, 13, 14, 15, and 17 if rated “true”. Higher scores (maximum = 17) reflect greater hopelessness or negative expectations towards the future.

Change in problem-solving ability at T3 and T4 when compared to T2 will be assessed using the Negative Problem Orientation Questionnaire (NPOQ) [50], which contains 12 items assessing dysfunctional attitudes related to problem-solving ability. Items are rated on a 5-point Likert scale from 1 (“not at all true of me”) to 5 (“extremely true of me”). Responses to each item are summed to calculate a total score, with a potential range of 12–60; higher scores indicate poorer problem-solving ability.

Change in current suicidal ideation at T2 when compared to T1 will be measured using a single purpose-designed item, “Are you thinking about killing yourself right now?”. If yes, participants will be asked to indicate which of the following describes the level of suicidal ideation they are experiencing: “Mild suicidal thoughts with no plan or intent to act”; “Moderate suicidal thoughts with a rough plan and some intent”; “Severe suicidal thoughts with a specific plan and intent to act”. The counts falling into the different levels will be used for analysis.

Change in health service use from formal and informal sources at T2, T3, and T4 when compared to T1 will be assessed using the Resource Use Questionnaire (RUQ) and a purpose-designed measure respectively. The RUQ was adapted from the Young Mind Matters Service Use questionnaire [51] and assesses use of mental health professionals and services, mental health-related hospitalisations, medication use for mental health reasons, and school-based mental health services. These data will also be used for the economic evaluation.

Change in attitudes towards seeking help from adults at school, and attitudes towards overcoming secrecy barriers, at T2, T3, and T4 when compared to T1, will be assessed using scales derived from the previous US Sources of Strength trial [52] and used in the Australian Sources of Strength trial [5]. Items are rated from 1 (strongly disagree) to 4 (strongly agree). Items on these scales are summed, with higher scores reflecting more positive attitudes towards help-seeking from adults at school, or more positive intentions to get help for suicidal friends and resist requests for secrecy.

Change in perceived ability and intention to intervene with a suicidal person at T2, T3, and T4 when compared to T1 will be assessed using the Perceived Behavioural Control and Intention subscales of the Willingness to Intervene Against Suicide Questionnaire (WIS) [53]. The scales have 20 and 22 items respectively. Both are rated on a 5-point scale ranging from 1 (“strongly disagree”) to 5 (“strongly agree”). Items 1, 3, 5, 10, 14, and 16 on the Perceived Behavioural Control subscale and items 12, 18, 19, 20, and 21 of the Intentions subscale are reverse-scored. The potential range of scores is 20–100 and 22–100 respectively; a higher score indicates a higher endorsement of intervening.

Change in stigma towards suicide at T2, T3, and T4 when compared to T1 will be assessed using the Stigma of Suicide Scale (SOSS)—Short Form [54]. This measure contains 16 one- or two-word descriptors of people who die by suicide, and respondents are asked to rate their level of agreement with each item on a 5-point scale ranging from 1 (“strongly disagree”) to 5 (“strongly agree”). The mean response to all items is calculated to score this instrument, with higher mean scores indicating greater suicide stigma.

Change in suicide literacy at T2, T3, and T4 when compared to T1 will be assessed using the Literacy of Suicide Scale (LOSS)—Short Form [55]. This scale contains 12 items rated on a “true/false/don’t know” scale. The correct response for items 1, 3, 4, 5, 7, 8, and 10 is “false” while items 2, 6, 9, 11, and 12 are correctly answered “true”. The scale provides a total literacy score (percent correct) where higher scores indicate greater suicide literacy.

Change in health-related quality of life during the course of the trial (T1, T2, T3, and T4) will be assessed using the Child Health Utility—9 Dimension (CHU9D) [56]. The CHU9D is a multi-attribute utility instrument suitable for young people aged 7–17 years. It comprises a short questionnaire alongside a set of preference weights using general population values. The questionnaire has nine items with five response levels per item and can be used to derive quality-adjusted life years (QALYs) for use in a cost-utility analysis (i.e. the economic evaluation).

#### Additional measures

To characterise the sample at baseline, participants will be administered a standard demographic questionnaire. They will also be asked about any previous mental health diagnoses, exposure to suicide in others, and any previous suicide prevention training undertaken.

At each time point, participants will be asked about their history of suicide planning and attempt, using the four suicide items from the Youth Risk Behaviour Survey (YRBS) [57]. These yes/no items assess if the respondent has had suicidal ideation or made a suicide plan or attempt (and its seriousness) during the past 12 months at baseline, or in the period between assessment points.

Participant views on the safeTALK workshop, including whether or not they thought it was “enjoyable”, “upsetting”, and “worthwhile”, will be assessed at T2 only using seven purpose-designed items. Participant views on the Reframe IT intervention (or for those in the control group, acceptability of *not* receiving Reframe IT) will be assessed using 11 purpose-designed items at T3 only.

Finally, three additional scales will be used that are not directly related to the aims of the present study. First, the Distress Questionnaire—5 (DQ-5) [58] will be administered at T1 to facilitate future comparison of data with another trial of a school-based suicide prevention programme in Australia [5]. The DQ-5 is a 5-item measure of distress, wherein participants are asked to specify on a 5-point scale, from 1 (“never”) to 5 (“always”), how frequently they have experienced certain indicators of distress over the past 30 days. The potential range of scores is 5–25, with higher scores indicating a greater level of distress. Next, two scales will be included as part of a larger study conducted by researchers in Europe examining whether participating in suicide prevention research is associated with distress. The first is the Patient Health Questionnaire—4 item version (PHQ-4) [59], a screener of anxiety and depression symptoms. As two of the items are included in the PHQ-9, only the remaining two items (“how often have you been bothered by feeling nervous, anxious or on edge”; “how often have you been bothered by not being able to stop or control worrying”) will be added to the T1 questionnaire only. The second scale is a purpose-designed 11-item scale to assess experimentally induced distress, which will be administered at T4. It includes two subscales: experimentally induced distress in absolute terms (i.e. participation was distressing) and in relative terms (i.e. participation was more distressing than everyday life events). This scale was adapted from Yeater, Miller, Rinehart, and Nason [60].

### Sample size and power calculation

The critical power calculation for this study relates to Component 2 (RCT of Reframe IT). The primary outcome variable for Reframe IT is reduced suicidal ideation, measured by the SIDAS, at 24 weeks (T4), in comparison to SIDAS scores at Time 2.

The Suicidal Ideation Questionnaire (SIQ) [61, 62] was used in the Reframe IT pilot study [33]. The SIQ and SIDAS are both continuous self-report measures of the frequency of cognitions related to suicide within the past month, with higher scores corresponding to more frequent and severe suicidal ideation. The range of the SIDAS is 0–50 and the range of the SIQ is 0–180. A SIDAS score of 1 or more indicates “risk for suicide”, whereas a SIQ score of 41 or more indicates “need for further evaluation of psychopathology”. As the SIQ licence is too expensive to use on a large scale, the SIDAS is being used in its place. In the absence of pilot data for the SIDAS, the power calculation is based on the SIQ. This can be reasonably justified given the similarities between the measures and their cut-offs and has been calculated in consultation with the study statistician.

In the Reframe IT pilot study, there were three time points of data collection (corresponding to T2, T3, and T4 as labelled in the current study). The mean reduction in SIQ between T1 and T3 (i.e. corresponding to T2 and T4 in the present study) for the pilot control group was 61.6 points (SD = 42.3) and for the pilot intervention group this was 47.1 points (SD = 41.6). The difference between these two reductions was 14.5 points. We also observed an intra-class correlation of 0.023 within schools, which gives a design effect of 1.1. Using these data, and making the conservative assumption that there is no correlation between SIQ scores within individuals over time, a 5% significance level and 80% power means that a sample size of 134 per arm (268 in total) is required.

**Fig. 1:**
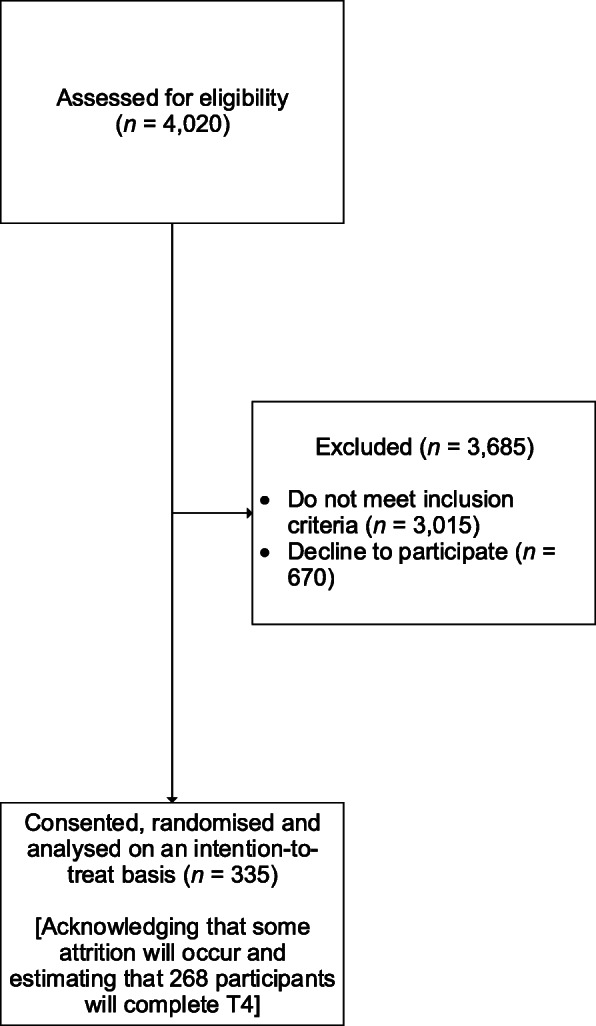
Sample size calculation based on projected rates of endorsement of suicidal ideation, consent, and attrition

Based on pilot data, and in consideration of the self-reported nature of the data collected, we estimate that, to recruit the required 268 students, we need to screen 4020 students (see Fig. 1). This is based on 20% drop-out rate among those who are classed as high-risk, consenting, and randomised; 33% of those identified as high-risk consenting to participate in the trial and being randomised (335 consenting and randomised, and analysed on an intention-to-treat basis); and 25% of the total sample endorsing suicidal ideation within the past month (1005 students). There are 141 mainstream secondary schools in the region and approximately 140 year 10 students per school. It is therefore estimated that approximately 29 schools will be recruited in total, although this number may fluctuate depending on the rate of student recruitment. Schools will be recruited in batches throughout the project. If the target sample size is reached before the 29 schools have been recruited, no further schools will be recruited. Similarly, if we recruit the target 29 schools but fail to recruit the required number of participants, more schools will be invited to participate. In the event that we struggle to meet the recruitment target, or if it is unfeasible to recruit year 10 students at a particular school, year 11 students may also be recruited.

Recruitment numbers will be monitored closely by the study team and the Sponsor. If it becomes apparent that we are not on track to meet our recruitment target, the study team will meet to discuss alternative recruitment measures. Recruitment rate success will be formally assessed every 6 months. The relevant ethics committees will be notified in the case of any proposed amendments to the protocol.

### Randomisation and treatment allocation

Students who consent to participate in Component 2 will be randomised to the intervention group (Reframe IT plus TAU) or to the control group (TAU only) using block randomisation with varying block sizes and stratified by school. The allocation ratio will be 1:1. The randomisation schedule is computer generated by independent Information Technology (IT) personnel with guidance from the study statistician. The schedule will then be implemented by a member of the study team (other than the statistician) into the REDCap database management software for allocating treatments to individual students.

A member of the study team will inform school wellbeing staff of the outcome of randomisation, and they will then inform students. With the exception of the study statistician, study team members are not blind to the treatment allocations. The study statistician will not have access to the randomisation component of the database and will not be told about the treatment allocations, and will therefore remain blinded. As the study statistician will not be involved in any aspect of this study operations other than data analysis, there are no circumstances we could foresee under which the study statistician would be required to be unblinded.

### Statistical analyses

#### Component 1

For each continuous outcome, changes over time will be assessed using linear mixed effects modelling with school included as a random factor. This analysis will examine whether there is a trend over time and whether the trend is linear or non-linear. Change in current suicidal ideation between T1 and T2 will be assessed using the McNemar–Bowker symmetry test for nominal data.

#### Component 2

Descriptive statistics will be used to compare participant characteristics between the study arms, and to assess for any imbalance.

The analysis will adopt the intention-to-treat approach, which is to include in the analysis all participants who have been randomised and regard each participant as belonging to the randomised group concerned irrespective of protocol adherence. Difference in change in the primary outcome of SIDAS score between the treatment groups will be examined using linear mixed effects modelling with the treatment group as a fixed factor and school included as a random factor. Secondary outcome measures will be similarly analysed. As a secondary analysis, compliance (as measured by the number of modules completed) and other potential covariates (e.g. gender) will be accounted for.

If the amount of missing data is non-trivial, multiple imputation will be considered to handle the missing data.

#### Economic evaluation

The economic evaluation will employ a within-trial design whereby the individual-level costs and outcomes of the randomised student participants will be analysed. A modelled economic evaluation will also be undertaken using the results of this trial and relevant epidemiological literature to extrapolate long-term costs and consequences that are not fully captured within the trial. The economic evaluation will comprise both a cost-utility analysis and a cost-consequences analysis. The cost-utility analysis will evaluate the cost-effectiveness of the intervention in terms of the cost per QALY, where costs are estimated using the RUQ and QALYs are measured using the CHU9D. A cost-consequences analysis will also be conducted to compare the incremental costs of the intervention to the full spectrum of outcomes included in the study (i.e. change in suicidal ideation, depressive symptoms, hopelessness, and problem-solving capability). This will produce a set of alternative cost-effectiveness ratios, an approach that has been deemed useful by decision-makers. The economic evaluation will be primarily conducted using the health sector perspective. A broader societal perspective will also be undertaken in a secondary analysis. The evaluation will first measure and value any change to the use of health care resources over the period of the study between the two arms of the trial (intervention and control) and then compare any additional costs to other outcomes achieved.

Standardised economic evaluation techniques including incremental analysis of mean differences and bootstrapping to determine confidence intervals will be employed. If the intervention is found to be effective, the lifetime and population budgetary impacts and cost-effectiveness of the intervention will be determined using modelling techniques.

### Safety, supervision, and committees

The organisational structure of this trial is as follows. The MAPSS steering committee is comprised of the CI, project manager, and clinical supervisor and is responsible for investigator oversight of the MAPSS project. The MAPSS steering committee meet on a fortnightly basis. The MAPSS Safety Monitoring Committee (SMC—Equivalent to the Data Safety and Management Committee (DSMC)) is the main vehicle for safety management, data monitoring, endpoint adjudication, and data management strategy. This committee is comprised of the CI, project manager, as well as independent internal and external subject matter experts including the Sponsor. The composition of the group was designed to incorporate transparency and so that no one set of competing interests could unduly influence other stakeholders, and is appropriate for this non-commercially funded or sponsored study. This committee has a dual safety role: it incorporates a risk-appropriate safety, endpoint adjudication, and data management strategy which is responsive to study issues as they eventuate. The committee meets on a quarterly basis and on an ad hoc basis when deemed necessary by the steering committee. The charter for this committee is outlined in the SMC manual, available upon reasonable request by contacting the corresponding author.

A comprehensive safety protocol has been developed, which will be activated if: (1) participants return a score of 1 or higher on the SIDAS at any time-point (the established cut-off [45] indicating any level of suicidal ideation in the past month); (2) participants report current suicidal ideation at any time-point; (3) participants report suicide risk via the Reframe IT platform. Where possible, the school will be responsible for checking in with the participant and managing any risk, although in some cases researchers will assist with this (e.g. where the school does not have the capacity to follow up with students in a timely way). If this is required, the researcher will contact the participant by phone and conduct a brief risk assessment. The response will depend on the level of risk, and ranges from providing the participant with contact information for helplines (in the case of low risk) to calling the participant’s emergency contact or emergency services (in the case of high-severe risk). All participants will be required to provide emergency contact information via the consent form. Ultimately, all risk information will be communicated to the school, who will be responsible for ongoing management. There are no planned interim analyses for this study, nor are there any stopping guidelines other than as outlined in section “Study discontinuation criteria” of this protocol.

Supervision will be provided to the researchers from the study clinical psychologist, who will also be on call during each assessment session.

### Adverse events

#### Definitions of adverse events and serious adverse events

##### Adverse event (AE)

An adverse event is the development of an untoward effect, undesirable clinical occurrence or medical condition, or the deterioration of a pre-existing medical condition following or during exposure to a study intervention, whether or not considered causally related to the study intervention. For the purposes of safety reporting, any research activity is considered to be part of the “study intervention”.

An AE can therefore be any unfavourable and unintended clinical sign (including an abnormal laboratory finding), symptom, observation, or disease temporally associated with the use of an intervention, whether or not related to the intervention.

##### Serious adverse event (SAE)

An SAE is any untoward medical occurrence that:

- Results in death;
- Is life-threatening;
  - Life-threatening in the definition of serious refers to an event in which the participant was at risk of death at the time of the event; it does not refer to an event which hypothetically might have caused death if it were more severe.
- Requires inpatient hospitalisation or prolongation of existing hospitalisation;
- Results in persistent or significant disability/incapacity;
- Is a congenital anomaly/birth defect;
- Is an important medical event that although not immediately life-threatening or result in death or hospitalisation, based upon appropriate medical and scientific judgement, may jeopardise the participant and/or require intervention to prevent one of the outcomes listed above.

Outpatient treatment in an emergency department is not in itself an SAE, although the reasons for it may be (e.g. suicide attempt).

Hospital admissions and/or surgical procedures planned before or during a study are not considered SAEs if the illness or disease existed (or the surgery was planned) before the participant was enrolled in the study, provided that it did not deteriorate in an unexpected way during the study.

#### Assessment and documentation of AEs

All AEs and SAEs that arise during the trial will be recorded in the study database. The causality of AEs and SAEs (i.e. their relationship to intervention treatment) will be assessed by a suitably qualified study team member. Where possible, information related to causality will be collected from the participant who experienced the adverse event (i.e. whether or not the participant themselves attributes the AE to the intervention) as well as any relevant collateral information from family members, friends, and school staff. Any AE that is serious (i.e. life-threatening) will be reported to the Sponsor and to the relevant ethics committees within 24 hours of the research team becoming aware of its occurrence, regardless of the relationship to the intervention.

### Confidentiality and data management

The PICF will explain that study data will be safely stored in computer databases as well as in paper form. The maintenance of confidentiality will be in accordance with national data and privacy legislation. Participants in this database will be identified by a unique participant identification number and their initials. The PICF will also explain that for data monitoring purposes, authorised representatives of Orygen, regulatory authorities, HRECs, or sites may require direct access to parts of the hospital or practice records relevant to the study, including medical history. The PICF will also explain that participants’ data from the two instruments that are included in the meta-study (PHQ-4 [59] and Yeater et al. [60] instrument) will be posted to the Open Science Framework public repository. No other participant data or identifying information of any kind will be posted to this repository. It will not be possible for the researchers or others to match data posted to the Open Science Framework repository to specific participants.

During the course of the study, any paper-based data will be securely stored in a locked filing cabinet on-site at Orygen.

Participant data will be anonymised and all electronic data files will be password-protected, accessible only by named members of the research team, and saved on encrypted University computers.

Participants’ data from the two instruments that are included in the meta-study (PHQ-4 and Yeater et al. instrument) will be posted to the Open Science Framework public repository. No other participant data or identifying information of any kind will be posted to this repository. It will not be possible for the researchers or others to match data posted to the Open Science Framework repository to specific participants.

Data collected automatically by the Reframe IT website is hosted in secure Amazon data centres located in Sydney, Australia. This data is encrypted using 256-bit Advanced Encryption Standard (AES-256). User passwords are encrypted using Bcrypt to ensure passwords are not stored in plain text. This data will be exported into an Excel file at least once annually and stored as described above.

All study documentation will be retained for a minimum of 15 years (following completion of the study) including participant files and other essential documents (study protocol, signed informed consent forms, correspondence, and other documents pertaining to the conduct of the study). Paper-based data will be destroyed through commercial confidential data disposal services. Electronic data will be deleted from the secure servers.

### Audits and Inspections

Authorised representatives of Orygen, a regulatory authority, or the HREC may visit the centre to perform audits or inspections. The CI will contact Orygen or the designee immediately if they are contacted about an inspection at their site. If an audit or inspection occurs, the CI and institution agree to allow the auditor/inspector direct access to all relevant documents and allocate their time and the time of their staff to the auditor/inspector to discuss findings and any relevant issues.

### Changes to the protocol

Study procedures will not be changed without the mutual agreement of the CI and Orygen.

If it is necessary for the study protocol to be amended, the amendment or a new version of the study protocol must be notified to or approved by the HREC before implementation unless the safety of participants is at risk. Local requirements must be followed.

If a protocol amendment requires a change to the PICF, approval of the revised PICF by Orygen and by the HREC is required before the revised form can be used to consent potential participants.

The trial registry and the journal in which the protocol is published will be notified of any protocol updates.

### Dissemination

Access to anonymised participant-level data will be granted upon reasonable request to the CI. All research findings, whether containing negative or positive results, will be disseminated accurately. These may be communicated via publication in academic journals and presentation at conferences. In addition, an aggregated report of study findings will be disseminated to all participating schools. Authorship for trial publications will follow the recommendations on authorship published by the International Committee of Medical Journal Editors [63]. The CI will be the arbiter of eligibility for authorship. There will be no usage of professional writers.

## Discussion

This study responds to the rising rates of suicide among young people in Australia but, as noted above, to date high-quality evidence is lacking in youth suicide prevention research [14]. In line with international best practice, this large-scale study will evaluate the efficacy and cost-effectiveness of a suicide prevention programme comprising universal, selective, and indicated components delivered in secondary school settings. We hypothesise that this programme will reduce suicide risk, improve risk recognition, and increase health service use among participants. The study is being conducted in partnership with the Department of Education in Victoria, so if found to be efficacious, safe, and cost-effective, the programme will not only contribute to a much-needed evidence-base, but would also have the potential to become embedded into standard school curricula and rolled out in school settings more broadly.

## Trial status

Recruitment of schools and participants was due to commence in February 2020. However, due to the COVID-19 pandemic, this will commence in February 2021.

## Supplementary Information

**Additional file 1.** Copy of original funding documentation.

**Additional file 2.** Approval from University of Melbourne Research Ethics Committee.

**Additional file 3.** Approval from Department of Education and Training Research Ethics Committee.

**Additional file 4.** SPIRIT Checklist.

**Additional file 5.** Participant Information and Consent Forms.

## Data Availability

All Principal Investigators will be given access to the cleaned data sets. Project data sets will be housed on the secure encrypted university server, and all data sets will be password protected. To ensure confidentiality, data dispersed to project team members will be blinded of any identifying participant information. Participants’ data from the two instruments that are included in the meta-study (PHQ-4 and Yeater et al. instrument) will be posted to the Open Science Framework public repository. No other participant data or identifying information of any kind will be posted to this repository. It will not be possible for the researchers or others to match data posted to the Open Science Framework repository to specific participants.

## Abbreviations

AE: Adverse event
AES-256: 256-bit Advanced Encryption Standard
ASIST: Applied Suicide Intervention Skills Training
CBT: Cognitive behavioural therapy
CI: Chief Investigator
CHU9D: Child Health Utility 9 Dimension
CONSORT: Consolidated Standards of Reporting Trials
DQ5: Distress Questionnaire - 5 item
DSMC: Data Safety and Monitoring Committee
HREC: Human Research Ethics Committee
HSC: Hopelessness Scale for Children
IT: Information Technology
LOSS: Literacy of Suicide Scale
MAPSS: Multimodal Approach to Preventing Suicide in Schools
NPOQ: Negative Problem Orientation Questionnaire
PICF: Participant Information and Consent Form
PHQ: Patient Health Questionnaire
QALY: Quality-adjusted life year
RUQ: Resource Use Questionnaire
SIDAS: Suicidal Ideation Attributions Scale
SIQ: Suicidal Ideation Questionnaire
SMC: Safety Monitoring Committee
SOSS: Stigma of Suicide Scale
SPIRIT: Standard Protocol Items: Recommendations for Interventional Trials
TAU: Treatment As Usual
WIS: Willingness to Intervene against Suicide
YRBS: Youth Risk Behaviour Scale
