# Supplementary material for: Study protocol for the Multimodal Approach to Preventing Suicide in Schools (MAPSS) project: a regionally based randomised trial of an integrated response to suicide risk among secondary school students
Source: Trials. 2022 Mar 2;23:186. doi: 10.1186/s13063-022-06072-8 (PMC8889397; doi:10.1186/s13063-022-06072-8)
Supplement: Supplementary file 1 — Additional file 1. Copy of original funding documentation. [file 13063_2022_6072_MOESM1_ESM.pdf]

Australian Government
National Health and Medical Research Council

NHMRC

GPO Box 1421 | Canberra ACT 2601
16 Marcus Clarke Street, Canberra City ACT 2600
T. 13 000 NHMRC (13 000 64672) or +61 2 6217 9000
F. +61 2 6217 9100
E. nhmrc@nhmrc.gov.au
ABN 88 601 010 284
www.nhmrc.gov.au

Doctor Jo Robinson
jo.robinson@orygen.org.au

Dear Doctor Robinson

********* UNDER EMBARGO AND PROVIDED IN CONFIDENCE *********

This advice and the document/s referred to below are provided under strict **embargo** and as such, on an In-Confidence basis. **The document/s and the information are not to be made public at this time by institutions or recipients**. NHMRC will notify your Administering Institution when your outcome is no longer under embargo.

****

Application ID: APP1153051
Type: Partnership Projects
Application Title: An integrated response to suicide risk among secondary schools: A regionally-based randomised trial

I am pleased to advise that the Minister for Health has approved your application (APP1153051) for National Health and Medical Research Council (NHMRC) Partnership Projects 2017 Third Call for funding commencing in 2018.

This letter provides you with important information about the offer of Funding made to University of Melbourne for this application.

Assessment Details

Where available[1], information about the assessment of your application is provided in a separate Application Assessment Summary. This can be accessed via RGMS following the instructions for accessing feedback in the *RGMS User Guide – Awarding Grants*.

Accepting this offer

The offer of Funding for your Application is made under NHMRC's Funding Agreement (the Funding Agreement) between the Australian Government and your Administering Institution. Your Administering Institution is responsible for informing you about the requirements of the Funding Agreement (including its Schedules) the Direct Research Costs guidelines and other applicable policies[2].

[1] An assessment summary is not available for applications to schemes where the NHMRC does not perform the peer review. Guidance on interpreting Project Grants Assessment Summaries is available at http://www.nhmrc.gov.au/grants/outcomes-funding-rounds.

[2] Copies of the Funding Agreement, and Direct Research Costs guidelines and other policies are available at http://www.nhmrc.gov.au/grants/administering-grants/nhmrc-funding-agreement

Your Administering Institution has until 31/05/2018 to certify that the information required prior to payment being made (see below) has been entered into RGMS, and to advise NHMRC of its acceptance of the offer. If the offer is not accepted by this date it may lapse. If you wish to discuss this offer of Funding, or have any queries, please contact your Research Administration Officer (RAO).

Information required prior to payment being made

Where applicable, and except where otherwise indicated, NHMRC will temporarily withhold some or all of the funding under subclause 15.2.a of the Funding Agreement with your Administering Institution until Specified Personnel with outstanding obligations from previous NHMRC grants, including submission of a Final Report, have met those obligations.

In some circumstances, CIAs and Fellows may need to provide additional ethics information. This information must be entered into RGMS by the CIA or Fellow and certified by the RAO. The *RGMS User Guide – Awarding Grants* provides details on how to enter and certify this data at award. Should you have any questions concerning the provision of such information, please speak to your RAO.

If you need to seek approval to defer the start date of this grant, please refer to Grantee Variations or speak with your RAO.

Funding

As set out in the Schedule to the Funding Agreement, the Partnership Projects APP1153051, has been awarded $666,281.00. Where applicable, this budget has been assessed by the expert peer review panel as sufficient to complete the aims and objectives of the research proposal stated in the application for funding. Any conditions relevant to receiving the Funding are set out in the Schedule to the Funding Agreement and, where applicable, the associated Funding Rules. All expenditure must be in accordance with the requirements of the Funding Agreement.

Participation in NHMRC Peer Review

NHMRC relies on the ongoing participation of the research community to ensure that every application receives expert peer review. NHMRC is grateful for this enormous contribution which is acknowledged through our website's peer review honour roll.

To ensure that applications for future rounds are appropriately assessed, we are reminding all Chief Investigators, Fellows and Scholars working on NHMRC Funded Research Activities that they may be requested to make themselves available to contribute to the peer review process, in accordance with clause 23.1 of the Funding Agreement.

Accordingly, we ask that you ensure your CV/Profile information is up to date in RGMS to assist in the identification of appropriate peer reviewers.

Yours sincerely

[Authorised for electronic transmission]

Dr Julie Glover
A/g Executive Director
Research Programs
