# Supplementary material for: Study protocol for the Multimodal Approach to Preventing Suicide in Schools (MAPSS) project: a regionally based randomised trial of an integrated response to suicide risk among secondary school students
Source: Trials. 2022 Mar 2;23:186. doi: 10.1186/s13063-022-06072-8 (PMC8889397; doi:10.1186/s13063-022-06072-8)
Supplement: Supplementary file 2 — Additional file 2. Approval from University of Melbourne Research Ethics Committee. [file 13063_2022_6072_MOESM2_ESM.pdf]

22 February 2019

A/Prof J.S. Robinson
Centre for Youth Mental Health
The University of Melbourne

Dear A/Prof Robinson

I am pleased to advise that the Psychology Health and Applied Sciences Human Ethics Sub-Committee has approved the following Project:

Project title: **The safeTALK and Reframe IT (STAR) project: A regionally-based randomised trial of an integrated response to suicide risk among secondary school students.**
Researchers: **Prof C Mihalopoulos, Prof J E Pirkis, Mr H P Yuen, A/Prof M J Spittal, Dr S M Rice, Miss E Bailey, A/Prof J S Robinson, Ms S Byrne, Ms M L Lamblin, Mr M Hamilton, A/Prof S E Hetrick, Ms N Stefanac, Ms A K Boland**
Ethics ID: **1852317**

The Project has been approved for the period: **22-Feb-2019 to 31-Dec-2019**

It is your responsibility to ensure that all people associated with the Project are made aware of what has actually been approved.

Research projects are normally approved to 31 December of the year of approval. Projects may be renewed yearly for up to a total of five years upon receipt of a satisfactory annual report. If a project is to continue beyond five years a new application will normally need to be submitted.

Please note that the following conditions apply to your approval. Failure to abide by these conditions may result in suspension or discontinuation of approval and/or disciplinary action.

(a) **Limit of Approval:** Approval is limited strictly to the research as submitted in your Project application.

(b) **Variation to Project:** Any subsequent variations or modifications you might wish to make to the Project must be notified formally to the Human Ethics Sub-Committee for further consideration and approval. If the Sub-Committee considers that the proposed changes are significant, you may be required to submit a new application for approval of the revised Project.

(c) **Incidents or adverse effects:** Researchers must report immediately to the Sub-Committee anything which might affect the ethical acceptance of the protocol including adverse effects on participants or unforeseen events that might affect continued ethical acceptability of the Project. Failure to do so may result in suspension or cancellation of approval.

(d) **Monitoring:** All projects are subject to monitoring at any time by the Human Research Ethics Committee.

(e) **Annual Report:** Please be aware that the Human Research Ethics Committee requires that researchers submit an annual report on each of their projects at the end of the year, or at the conclusion of a project if it continues for less than this time. Failure to submit an annual report will mean that ethics approval will lapse.

(f) **Auditing:** All projects may be subject to audit by members of the Sub-Committee.

If you have any queries on these matters, or require additional information, please contact me using the details below. Please quote the ethics ID number and the title of the Project in any future correspondence. On behalf of the Sub-Committee I wish you well in your research.

Yours sincerely

Mr Tony Callahan
Secretary, Psychology Health & Applied Sciences HESC
Phone: 8344 2017, Email: t.callahan@unimelb.edu.au
