# Supplementary material for: Study protocol for the Multimodal Approach to Preventing Suicide in Schools (MAPSS) project: a regionally based randomised trial of an integrated response to suicide risk among secondary school students
Source: Trials. 2022 Mar 2;23:186. doi: 10.1186/s13063-022-06072-8 (PMC8889397; doi:10.1186/s13063-022-06072-8)
Supplement: Supplementary file 3 — Additional file 3. Approval from Department of Education and Training Research Ethics Committee. [file 13063_2022_6072_MOESM3_ESM.pdf]

Department of Education & Training

2 Treasury Place
East Melbourne Victoria 3002
Telephone: 03 9637 2000
DX210083

2019_003951

Associate Professor Jo Robinson
Orygen, The National Centre of Excellence in Youth Mental Health
The University of Melbourne
Locked Bag 10, 35 Poplar Road
PARKVILLE 3052

Dear Associate Professor Robinson

Thank you for your application of 31 January 2019 in relation to your work which has been commissioned by the Department of Education and Training to conduct research in Victorian government schools titled *The safeTALK and Reframe IT (STAR) Project: A regionally-based randomised trial of an integrated response to suicide risk among secondary school students.*

I am pleased to advise that on the basis of the information you have provided your research proposal is approved in principle subject to the conditions detailed below.

1. Department approved research projects currently undergoing a Human Research Ethics Committee (HREC) review are required to provide the Department with evidence of the HREC approval once complete.

2. The research is conducted in accordance with the final documentation you provided to the Department of Education and Training.

3. Separate approval for the research needs to be sought from school principals. This is to be supported by the Department of Education and Training approved documentation and, if applicable, the letter of approval from a relevant and formally constituted HREC.

4. The project is commenced within 12 months of this approval letter and any extensions or variations to your study, including those requested by an ethics committee must be submitted to the Department of Education and Training for its consideration before you proceed.

5. As a matter of courtesy, you advise the relevant Regional Director of the schools that you intend to approach. An outline of your research and a copy of this letter should be provided to the Regional Director.

6. You acknowledge the support of the Department of Education and Training in any publications arising from the research.

Your details will be dealt with in accordance with the *Public Records Act 1973* and the *Privacy and Data Protection Act 2014*. Should you have any queries or wish to gain access to your personal information held by this department please contact our Privacy Officer at the above address.

VICTORIA State Government

7. The Research Agreement conditions, which include the reporting requirements at the conclusion of your study, are upheld. A reminder will be sent for reports not submitted by the study's indicative completion date.

I wish you well with your research. Should you have further questions on this matter, please contact Youla Michaels, Project Support Officer, Insights and Evidence Branch, by telephone on (03) 7022 0306 or by email at michaels.youla.y@edumail.vic.gov.au.

Yours sincerely

Joyce Cleary
Director
Insights and Evidence

29/03/2019

VICTORIA
State
Government
