# Supplementary material for: Study protocol for the Multimodal Approach to Preventing Suicide in Schools (MAPSS) project: a regionally based randomised trial of an integrated response to suicide risk among secondary school students
Source: Trials. 2022 Mar 2;23:186. doi: 10.1186/s13063-022-06072-8 (PMC8889397; doi:10.1186/s13063-022-06072-8)
Supplement: Supplementary file 5 — Additional file 5. Participant Information and Consent Forms. [file 13063_2022_6072_MOESM5_ESM.pdf]

Plain Language Statement

Centre of Youth Mental Health, University of Melbourne & Orygen

**Dated:**	09/06/2020
**Site:**	Orygen
**Full project title:**	Multimodal Approach to Preventing Suicide in Schools (MAPSS): A regionally-based trial of safeTALK and Reframe IT among secondary school students.
**Project phase:**	1 (evaluation of safeTALK)
**Responsible researcher:**	Associate Professor Jo Robinson
	Email: jo.robinson@orygen.org.au
**Associate researchers:**	Professor Jane Pirkis, Professor Cathy Mihalopoulos, A/Professor Matthew Spittal, Dr Simon Rice, A/Professor Sarah Hetrick, Matthew Hamilton, Hok Pan Yuen, Michelle Lamblin, Sadhbh Byrne, Eleanor Bailey, Meaghan Dickens, Alexandra Boland, Karolina Krysinska, Alison Clarke, Louise La Sala, India Bellairs-Walsh, Caitlin Bleeker, Nicole Hill, Katrina Witt, Pinar Thorn, Zoe Teh.
**Funding sources:**	The National Health and Medical Research Council

Introduction

Thank you for your interest in our research project. Here you'll find more information about the project, so that you can decide if you would like to take part.

Please read this information carefully. You can ask questions about anything you don't understand or want to know more about.

Your participation in this study is voluntary and there will be no cost to you. If you do not want to take part in this study you do not have to. Choosing not to take part in this study will not affect your current and future medical care in any way. You can also stop at any time.

What is this research about?

Unfortunately, suicidal thoughts and behaviours are common in young people. Therefore, schools are an important place for running suicide prevention programs, and

research tells us a number of activities can help. These include learning about the warning signs for suicide and how to find help, identifying students who are thinking about suicide, and individual therapy (e.g., sessions with the school counsellor). Research also tells us that combining different suicide prevention activities into large programs might be the best way to help young people experiencing suicidal thoughts and behaviours.

This research project is run by researchers at Orygen. It will test a few different types of suicide prevention activities in schools across North-West Melbourne. This consent form relates to one of these activities, an educational suicide prevention workshop called safeTALK.

safeTALK is a three-hour workshop designed to teach you how to: 1) recognise warning signs for suicide in others, 2) have a conversation with someone you are worried about, and 3) help that person access help. A small study by our team in 2015-2016 found that school students who completed the safeTALK workshop felt more confident about recognising and responding to warning signs in themselves and their friends. We also found that safeTALK did not make students feel very upset or suicidal.

The aim of this part of the project is to evaluate the safeTALK program with a much larger group of students. It will examine whether or not completing safeTALK can:

1. Increase your knowledge about suicide and suicide prevention;
2. Increase the likelihood of you seeking help;
3. Increase the likelihood of you feeling able to help others.

We will also examine whether or not safeTALK is acceptable to or causes distress in high school students.

We aim to recruit approximately 4,020 students from different schools in Melbourne to participate.

What will I be asked to do?

You are invited to participate in this study because you are a student in either year ten or year eleven at a school in North-West Melbourne, and your school has agreed to take part in the research project. If you agree to participate, you will complete a survey at four (4) time points, each of which will take approximately 35 minutes to complete (in class time). About two weeks after the first survey (1), you will receive the safeTALK training (3hr workshop, during class time). This will happen at your school in groups of about thirty students. Immediately after the safeTALK training, you will complete a survey again (2). You will then complete a survey ten weeks later (3), and again twelve weeks after that (4). The surveys will be mostly the same each time, with a few small differences.

The surveys will include questions about your:

- demographic information;
- experience of safeTALK training;
- knowledge about suicide prevention;
- experience of depressed feelings, coping and help-seeking;
- attitudes towards help-seeking and suicide, and willingness to help if someone you knew was thinking about suicide; and
- there will also be some questions about your own experience of suicidal thoughts and behaviour.

<u>If we are worried about you based on your responses to these questions, we will have to share these concerns with your school counsellor</u>, who will check in with you. That is, if your survey responses indicate that you are thinking about suicide or may be at risk, your school counsellor will be informed in order to link you with appropriate support. **<u>Please note: any decision taken by the school to notify your emergency contact and/or parent or guardian of your suicide risk, will occur according to the school's existing risk management protocols</u>.** In some cases, a member of the research team might also check in with you, by phone or in person, to check that you are ok. This will be the case each time you complete the survey.

Your answers to questions in the first survey may qualify you to participate in a second component of the study. In this case, a member of the research team will contact you, either by phone or in person to provide you with more information about the second component of the study, and check if you are interested in taking part. All the information required to help you decide whether to take part in the second component of the study will be outlined in a separate consent form, and explained to you, at that time.

If you consent to the study, but miss the first survey, you may be given the opportunity to do this survey any time up to the date of the safeTALK workshop. If you fail to complete the first survey before the workshop, you will be ineligible to participate in the study. If you arrive more than 30 minutes late to the safeTALK training you will not be able to complete it, as you will have missed important safety and self-care information delivered at the start of the training.

In most cases you will complete each of the surveys online, either using an iPad provided by the research team, your own tablet or computer, or one at your school. In some cases, you may be required to complete a paper version (e.g., if there are technical issues or no devices available). You will be allowed to complete the surveys at home electronically if you are unable to finish during the allotted time.

The total expected duration of your participation in this project is over a period of 6 months.

What are the possible benefits?

We hope that participating will help participants to identify suicide risk in themselves and their friends, have safe conversations about suicide, and know where to go for help. Another possible benefit is that young people who feel distressed or suicidal can be linked in with the school counsellor or other appropriate services. However, we cannot guarantee any benefits from taking part. What we do hope is that the information that we collect will allow us to help other young people in the future.

What are the possible risks?

There are no physical risks associated with being in this study; however, there may be unforeseen or unknown psychological risks. If you feel upset while filling in surveys or participating in the safeTALK training, please tell a member of the research team or school staff. If necessary, they can arrange additional support for you.

Do I have to take part?

No. Participation is completely voluntary. If you don't want to take part, you don't have to. If you decide to take part, you will be given this Participant Information and Consent Form to sign, and you will be given a copy to keep.

What if I want to withdraw from the research study?

If you do consent to participate, you may withdraw at any time. If you do withdraw, you will be asked to complete and sign the 'Revocation of Consent Form' which is provided at the end of this document. Alternatively, you can ring the research team and tell them you no longer want to participate. If you decide to leave the research study, the researchers will not collect additional information from you. Please let us know at the time when you withdraw what you would like us to do with the information we have collected about you up to that point. If you wish, your information will be removed from our study records and will not be included in the study results, up to the point that we have analysed and published the results.

If you withdraw from the study but do not ask for your data collected thus far to be removed, the data will continue to be used by the researchers and will be retained as described above. Your privacy will continue to be protected at all times.

Your decision about taking part, or to withdraw, will not in any way affect your relationship with Orygen or the University of Melbourne.

Will I hear about the results of this project?

Results from the study will be made available to participating schools in an annual progress report that may be shared via the school's newsletter, or presentations to staff,

parents and students. Results of the larger trial will be published in academic journals. We will provide copies of the published results to you if you would like. In any publication and/or presentation, information will be provided in such a way that you cannot be identified.

What will happen to information about me?

By signing this Consent Form, you agree to the research staff collecting and using personal information about you for this research project. Any information obtained that can identify you will be treated as confidential and securely stored. It will be disclosed only with your permission, or to a school counsellor if we become concerned about your safety, or as required by law.

During this study, all of your records will be kept strictly confidential (including electronic information). This means that only the Investigator(s) and research staff directly involved in this project will have access to them. The records will be kept in a locked office at Orygen. Electronic copies of confidential information will be stored on secure servers and will be password protected and accessed only by the researchers involved in this project. Your data will have a unique code, which will be linked to your contact details, which is kept in a separate password-protected file, for the purpose of contacting you for further information or, with your consent, contacting you for future research projects. Only study team members will have access to the link between the unique code and your contact details.

However, if we are concerned about risk to you or to someone else, we will need to take all reasonable steps to ensure you are safe and will therefore need to tell your school counsellor.

Your study data may be stored in the Orygen Cloud. "In the Orygen Cloud" refers to servers in a data centre that are managed by a third party and accessible through the Internet. When storing your study data, we will replace your name with a unique code on all your study data. The coded data will be encrypted and stored on a secure Cloud server to prevent improper access. This data will be part of a bigger Orygen database, and if you agree, we would like to be able to use your data to contribute to other research. All data collected from you will be kept for at least 25 years, but may be kept indefinitely.

You will be asked to provide additional consent for the use of your data for future research. The use of your data for future research is optional, so you do not have to consent to it if you don't want to, while still taking part in this study. Future research projects may be closely related or unrelated to this research project. It is unlikely that these studies will have a direct benefit to you, and you will not receive results from these future research projects. Any future research projects wanting to use your data will have to be reviewed and approved by a recognised Human Research Ethics Committee.

You will also be asked to provide additional consent for the use of your data in a separate larger international study of distress in suicide and self-harm research, led by a team of researchers at Ghent University and KU Leuven. The aim of this larger study is to examine whether distress is associated with participation in suicide and self-harm research. Only two sets of questions (the first in survey one, the second in survey four) will be used for this larger study, and will be clearly labelled in the surveys. If you allow your data to be used for this larger study it will be posted to the Open Science Framework public repository. This is a website that is designed to help researchers collaborate on specific projects by securely sharing data. No other data about you or identifying information will be posted to this repository. It will not be possible for anyone to identify this data as yours.

Who is funding this project?

This project is funded by a National Health and Medical Research Council (NHMRC) project grant, in partnership with Lifeline Australia and The Victorian Department of Education and Training.

Where can I get further information?

If you would like more information about the project, please contact the researchers at mapssproject@orygen.org.au.

Who can I contact if I have any concerns about the project?

This research project has been approved by the Human Research Ethics Committee of The University of Melbourne. If you have any concerns or complaints about the conduct of this research project, which you do not wish to discuss with the research team, you should contact the Manager, Human Research Ethics, Research Ethics and Integrity, University of Melbourne, VIC 3010. Tel: +61 3 8344 2073 or Email: HumanEthics-complaints@unimelb.edu.au. All complaints will be treated confidentially. If you contact the ethics committee about this project, please provide the name of the research team or the name or ethics ID number of the research project.

STUDENT TO SIGN

Participant Consent Form

Centre of Youth Mental Health, The University of Melbourne & Orygen

THE UNIVERSITY OF MELBOURNE

**Dated:**	09/06/2020
**Site:**	Orygen
**Full project title:**	Multimodal Approach to Preventing Suicide in Schools (MAPSS): A regionally-based trial of safeTALK and Reframe IT among secondary school students.
**Project phase:**	1 (evaluation of safeTALK)
**Responsible researcher:**	Associate Professor Jo Robinson
	Email: jo.robinson@orygen.org.au
**Associate researchers:**	Professor Jane Pirkis, Professor Cathy Mihalopoulos, A/Professor Matthew Spittal, Dr Simon Rice, A/Professor Sarah Hetrick, Matthew Hamilton, Hok Pan Yuen, Michelle Lamblin, Sadhbh Byrne, Eleanor Bailey, Meaghan Dickens, Alexandra Boland, Karolina Krysinska, Alison Clarke, Louise La Sala, India Bellairs-Walsh, Caitlin Bleeker, Nicole Hill, Katrina Witt, Pinar Thorn, Zoe Teh.
**Funding sources:**	The National Health and Medical Research Council

1. I consent to participate in this project, the details of which have been explained to me, and I will be provided with a written plain language statement to keep.

2. I understand that the purpose of this research is to investigate the impact of the safeTALK program.

3. I understand that the researchers may communicate with my school wellbeing team if they are worried about my safety, and that any action taken by my School to contact my parent/guardian about concerns for my safety will occur according to the School's existing risk management policy.

4. I understand that my participation in this project is for research purposes only.

5. I acknowledge that the possible effects of participating in this research project have been explained to my satisfaction.

6. In this project I will be required to complete a survey at four time points. Two weeks after the first time point, I will participate in the safeTALK training. If I miss the first timepoint of the study, baseline at Time 1, I will not be able to participate in the SafeTALK training or the remainder of the study.

7. I understand that my participation is voluntary and that I am free to withdraw from this project anytime without explanation or prejudice and to withdraw any unprocessed data that I have provided.

8. I understand that the data from this research will be stored at Orygen for at least 25 years but may be kept indefinitely.

9. I have been informed that the confidentiality of the information I provide will be safeguarded subject to any legal requirements; my data will be password protected and accessible only by the named researchers.

10. I understand that after I sign and return this consent form, it will be retained by the researcher.

Signature of Student Participant

Name of student participant (please print): ______________________________

Student school email address: ______________________________

Mobile Number: ______________________________

Signature of Student Participant: ____________________ Date: ______________

Emergency Contact Information*

Name of emergency contact: ______________________________

Relationship to you: ______________________________

Emergency contact's phone number: ______________________________

**we will use this information only if absolutely necessary (i.e., we are worried for your safety)*

OPTIONAL CONSENT (please tick)

☐ I agree to my de-identified information being used for future research

☐ I agree to a small portion of my de-identified information being shared with an international data repository (larger international study – see page 5 of this form).

☐ I do not agree to either of the above

Participant's Signature: ____________________ Date: ______________

FOR RESEARCHER:

Name of Researcher (please print) ______________________________

Signature ____________________ Date ______________

Note: All parties signing the consent section must date their own signature.

PARENT/GUARDIAN TO SIGN

Third Party Consent Form

Centre of Youth Mental Health, The University of Melbourne & Orygen

THE UNIVERSITY OF MELBOURNE

**Dated:**	09/06/2020
**Site:**	Orygen
**Full project title:**	Multimodal Approach to Preventing Suicide in Schools (MAPSS): A regionally-based trial of safeTALK and Reframe IT among secondary school students.
**Project phase:**	1 (evaluation of safeTALK)
**Responsible researcher:**	Associate Professor Jo Robinson
	Email: jo.robinson@orygen.org.au
**Associate researchers:**	Professor Jane Pirkis, Professor Cathy Mihalopoulos, A/Professor Matthew Spittal, Dr Simon Rice, A/Professor Sarah Hetrick, Matthew Hamilton, Hok Pan Yuen, Michelle Lamblin, Sadhbh Byrne, Eleanor Bailey, Meaghan Dickens, Alexandra Boland, Karolina Krysinska, Alison Clarke, Louise La Sala, India Bellairs-Walsh, Caitlin Bleeker, Nicole Hill, Katrina Witt, Pinar Thorn, Zoe Teh.
**Funding sources:**	The National Health and Medical Research Council

1. I give my permission for ______________________________ to participate in this project and I will be provided with a written plain language statement to keep.
2. I understand that the purpose of this research is to investigate the impact of the safeTALK program.
3. I understand that the researchers may communicate with the school wellbeing team if they are worried about the safety of my child, and that any action taken by the School to contact me about concerns for my child's safety will occur according to the School's existing risk management policy.
4. I understand that my child's participation in this project is for research purposes only.
5. I acknowledge that the possible effects of participating in this research project have been explained to my satisfaction.
6. I understand that my child will be required to complete a survey at four time points. Two weeks after the first time point they will participate in the safeTALK training.

7. I understand that my child's participation is voluntary and that they are free to withdraw from this project anytime without explanation or prejudice and to withdraw any unprocessed data that they have provided.

8. I understand that the data from this research will be stored at Orygen indefinitely.

9. I have been informed that the confidentiality of the information my child provides will be safeguarded subject to any legal requirements; their data will be password protected and accessible only by the named researchers.

10. I understand that after I sign and return this consent form, it will be retained by the researcher.

OPTIONAL CONSENT (please tick)

☐ I agree to my child's de-identified information being used for future research
☐ I agree to a small portion of my child's de-identified information being shared with an international data repository (larger international study – see page 5).
☐ I do not agree to either of the above

Signature of Parent or Guardian

Name of student participant (please print) ______________________

Name of parent or guardian ______________________

Relationship to participant ______________________

Signature of parent or guardian ______________________ Date ____________

Signature of Researcher

Name of Researcher (please print) ______________________

Signature ______________________ Date ____________

Note: All parties signing the consent section must date their own signature.

Signature of Witness (ONLY IF THE PARENT/GUARDIAN CANNOT READ THIS FORM THEMSELVES)

Name of Witness (please print) ______________________

Signature ______________________ Date ____________

Plain Language Statement

Centre of Youth Mental Health, University of Melbourne & Orygen

THE UNIVERSITY OF MELBOURNE

**Dated:**	09/06/2020
**Site:**	Orygen
**Full project title:**	Multimodal Approach to Preventing Suicide in Schools (MAPSS): A regionally-based trial of safeTALK and Reframe IT among secondary school students
**Project phase:**	2 (evaluation of Reframe IT)
**Responsible researcher:**	Associate Professor Jo Robinson
	Email: jo.robinson@orygen.org.au
**Associate researchers:**	Professor Jane Pirkis, Professor Cathy Mihalopoulos, A/Professor Matthew Spittal, Dr Simon Rice, A/Professor Sarah Hetrick, Matthew Hamilton, Hok Pan Yuen, Michelle Lamblin, Sadhbh Byrne, Eleanor Bailey, Meaghan Dickens, Alexandra Boland, Karolina Krysinska, Alison Clarke, Louise La Sala, India Bellairs-Walsh, Caitlin Bleeker, Nicole Hill, Katrina Witt, Pinar Thorn, Zoe Teh.
**Funding sources:**	The National Health and Medical Research Council

Introduction

Thank you for your interest in participating in our research project. Here you will find further information about the project, so that you can decide if you would like to take part.

Please read this information carefully. You can ask questions about anything you don't understand or want to know more about.

Your participation in this study is voluntary and there will be no cost to you. If you do not want to take part in this study you do not have to. Choosing not to take part in this study will not affect your current and future medical care in any way. You can also stop at any time.

What is this research about?

Unfortunately, suicidal thoughts and behaviours are common in young people. Therefore, schools are an important place for running suicide prevention programs, and research tells us a number of activities can help. These include learning about the warning signs for suicide and how to find help, identifying students who are thinking about suicide, and individual therapy (e.g., sessions with the school counsellor). Research also tells us that combining different suicide prevention activities into large programs might be the best way to help young people experiencing suicidal thoughts and behaviours.

This research project is run by researchers at Orygen. It will test a few different types of suicide prevention activities in schools across North-West Melbourne. This consent form relates to one of these activities, an online program for people who might experience suicidal thoughts. The program is called Reframe IT.

Reframe IT is a website designed to help young people who feel depressed or overly worried about things, or who feel so sad and hopeless that they think about dying. It includes eight 20-minute modules designed to be completed over eight weeks (i.e., one module per week). The modules include video diaries of young people talking about their experiences of feeling down, and a 'host' who uses their experiences to talk about different ways of coping with difficult thoughts or feelings. The young people on Reframe IT are actors, but the issues they talk about are issues that young people often experience. Each Reframe IT module also includes two activities. Reframe IT is checked once a day during weekdays by member of the research team, or "moderator", to monitor your progress and answer any questions you might have. Reframe IT therefore includes a message board for you to contact the moderator. You can also use the message board to record your feelings if you would like to.

It is important to note that Reframe IT is not designed to respond in an emergency. Therefore, if you are feeling very upset or suicidal it is important you talk to your school counsellor, or another trusted adult. You can also ring Lifeline on 13 11 14 (available 24/7), Kids Helpline on 1800 55 1800 (available 24/7), or Orygen Youth Health triage: 1800 888 320 (available 24/7). You can also access e-headspace online at https://www.eheadspace.org.au/ (available 9am-1am Melbourne time, every day).

Our team has run two studies testing Reframe IT, and we found the young people who used it reported improvements in their mental health and problem-solving skills. The young people who participated in these studies also said that they liked Reframe IT and would recommend it to other young people. However, these two previous studies had a small number of participants. Therefore, the aim of the current project is to evaluate the Reframe IT program with a much larger group of participants. We are aiming to recruit approximately 268 students to participate in this part of the project.

This part of the project will examine whether or not Reframe IT can help to reduce suicidal thoughts, symptoms of depression and feelings of hopelessness. We are also interested in whether or not Reframe IT can increase coping skills and help-seeking.

We are testing Reframe IT using a "randomised controlled design". This means that only half the people who agree to participate will be given access to the Reframe IT website, and the other half will receive "treatment as usual", which just means whatever counselling or support is usually provided by your school wellbeing team or outside of school. This is so we can compare the two groups to check if Reframe IT actually works.

What will I be asked to do?

You are already participating in another component of this project, the safeTALK evaluation, and you have completed the first survey. You have been invited to participate in this component of the project based on your responses to that survey.

If you agree to participate in this component of the project, you will be randomly assigned to either the 'treatment' or 'control' group using a computer program – this means that you have an equal chance of being in either group.

If you are in the treatment group, you will be provided with an ID and secure password which will allow you to access Reframe IT online. You will then be asked to complete one module each week for eight weeks. You will need to complete the modules in or near your school counsellor's office, in case you have any questions or feel upset while using Reframe IT. The timing of these sessions will be decided between you and your school counsellor. You will also continue to receive any other mental health treatment you would usually receive in addition to using Reframe IT.

If you are allocated to the control group you will continue to receive the same level of support as you would normally (e.g., from the school counsellor, from headspace, etc.).

Because you are already participating in the safeTALK evaluation and completing a survey at four time-points, you do not need to complete any additional surveys or questionnaires for this component of the project. The only exception to this is that there will be a few extra questions to answer specifically about your experience with Reframe IT in the third questionnaire only.

What are the possible benefits?

Possible benefits for both the treatment and control group are as follows:

- If you are seeing your school counsellor, or any other mental health professional and you are happy for us to do so, we can provide information to this person **from your survey responses**. This may help you to get better treatment.

- If you are not being seen **regularly** by your school counsellor or another mental health professional, participating in this project will mean your general psychological **wellbeing** will be checked over the 6 months of the project. If during the project you become unwell, project staff will be able to help you to get more help if you would like them to.

Additional benefits for the treatment group:

- You will have access to the interactive website, which we hope you will find helpful in some way.
- You will also be asked to complete a weekly screen to check whether or not you are feeling suicidal. This will be monitored weekly by the school counsellor.
- You will have the opportunity to give your opinion on ways in which the website could be improved.

However, we cannot guarantee any benefits from you taking part. What we do hope is that the information that we collect will allow us to be better able to help young people in the future.

What are the possible risks?

There are no physical risks associated with being in this study; however, there may be unforeseen or unknown psychological risks. If you feel upset while filling in questionnaires or using the Reframe IT website, please tell your school counsellor. If necessary, they can arrange additional support for you.

Do I have to take part?

No. Participation is completely voluntary. If you don't want to take part, you don't have to. If you decide to take part, you will be given this Participant Information and Consent Form to sign and you will be given a copy to keep.

What if I want to withdraw from the research study?

If you do consent to participate, you may withdraw at any time. If you do withdraw, you will be asked to complete and sign the 'Revocation of Consent Form' which is provided at the end of this document. Alternatively, you can ring the research team and tell them you no longer want to participate. If you decide to leave the research study, the researchers will not collect additional information from you. Please let us know at the time when you withdraw what you would like us to do with the information we have collected about you up to that point. If you wish, your information will be removed from our study records and will not be included in the study results, up to the point that we have analysed and published the results.

If you withdraw from the study but do not ask for your data collected thus far to be removed, the data will continue to be used by the researchers and will be retained as described above. Your privacy will continue to be protected at all times.

Your decision whether to take part or not to take part, or to take part and then withdraw, will not affect in any way your relationship with Orygen or the University of Melbourne.

Will I hear about the results of this project?

Results from the study will be made available to participating schools in an annual progress report that may be shared via the school's newsletter, or presentations to staff, parents and students. Results of the larger trial will be published in academic journals. We will provide copies of the published results to you if you would like. In any publication and/or presentation, information will be provided in such a way that you cannot be identified.

What will happen to information about me?

Information collected in the surveys will be treated as described in first consent form, which you have already signed.

If you participate in this component of the project, the following information will also be collected from you: 1) your answers to the questions specifically about Reframe IT, included in the third survey; 2) data collected automatically by the website such as number of log-ins and any information you enter in the activities or message board (only applicable if you are assigned to the treatment group).

By signing the Consent Form you consent to the research staff collecting and using this information for this research project. This data will be used in the same way as data collected in component 1 will be used. This has been repeated below:

During this study, all of your records will be kept strictly confidential (including electronic information). This means that only the Investigator(s) and research staff directly involved in this project will have access to them. The records will be kept in a locked office at Orygen. Electronic copies of confidential information will be stored on secure servers and will be password protected and accessed only by the researchers involved in this project. Your data will have a unique code, which will be linked to your contact details, which is kept in a separate password-protected file, for the purpose of contacting you for further information or, with your consent, contacting you for future research projects. Only study team members will have access to the link between the unique code and your contact details.

However, if we are concerned about risk to you or to someone else, we will need to take all reasonable steps to ensure you are safe and will therefore need to tell your school counsellor.

Your study data may be stored in the Orygen Cloud. "In the Cloud" refers to servers in a data centre that are managed by a third party and accessible through the Internet. When storing your study data, we will replace your name with a unique code on all your study data. The coded data will be encrypted and stored on a secure Cloud server to prevent improper access. This data will be part of a bigger Orygen database, and if you agree, we would like to be able to use your data to contribute to other research

All data collected from you will be kept for at least 25 years but may be kept indefinitely**.** After this time, paper copies of consent forms will be shredded, making re-identification of the data impossible.

You will be asked to provide additional consent for the use of your data for future research. The use of your data for future research is optional, so you do not have consent to it if you don't want to, while still taking part in this study. Future research projects may be closely related or unrelated to this research project. It is unlikely that these studies will have a direct benefit to you, and you will not receive results from these future research projects. Any future research projects wanting to use your data will have to be reviewed and approved by a recognised Human Research Ethics Committee.

Who is funding this project?

This project is funded by a National Health and Medical Research Council (NHMRC) grant, in partnership with Lifeline Australia and The Victorian Department of Education and Training.

Where can I get further information?

If you would like more information about the project, please contact the researchers at mapssproject@orygen.org.au.

Who can I contact if I have any concerns about the project?

This research project has been approved by the Human Research Ethics Committee of The University of Melbourne. If you have any concerns or complaints about the conduct of this research project, which you do not wish to discuss with the research team, you should contact the Manager, Human Research Ethics, Research Ethics and Integrity, University of Melbourne, VIC 3010. Tel: +61 3 8344 2073 or Email: HumanEthics-complaints@unimelb.edu.au. All complaints will be treated confidentially. If you contact the ethics committee about this project please provide the name of the research team or the name or ethics ID number of the research project.

STUDENT TO SIGN

Consent Form

Centre of Youth Mental Health, University of Melbourne & Orygen

THE UNIVERSITY OF MELBOURNE

**Dated:**	09/06/2020
**Site:**	Orygen
**Full project title:**	The safeTALK and Reframe IT (STAR) project: A regionally-based randomised trial of an integrated response to suicide risk among secondary school students.
**Project phase:**	2 (evaluation of Reframe IT)
**Responsible researcher:**	Associate Professor Jo Robinson
	Email: jo.robinson@orygen.org.au
**Associate researchers:**	Professor Jane Pirkis, Professor Cathy Mihalopoulos, A/Professor Matthew Spittal, Dr Simon Rice, A/Professor Sarah Hetrick, Matthew Hamilton, Hok Pan Yuen, Michelle Lamblin, Sadhbh Byrne, Eleanor Bailey, Meaghan Dickens, Alexandra Boland, Karolina Krysinska, Alison Clarke, Louise La Sala, India Bellairs-Walsh, Caitlin Bleeker, Nicole Hill, Katrina Witt, Pinar Thorn, Zoe Teh.
**Funding sources:**	The National Health and Medical Research Council

Name of Participant:

1. I consent to participate in this project, the details of which have been explained to me, and I have been provided with a written plain language statement to keep.
2. I understand that the purpose of this research is to investigate the impact of the Reframe IT website.
3. I understand that my participation in this project is for research purposes only.
4. I acknowledge that the possible effects of participating in this research project have been explained to my satisfaction.
5. I understand that my participation is voluntary and that I am free to withdraw from this project anytime without explanation or prejudice and to withdraw any unprocessed data that I have provided.
6. I understand that the data from this research will be stored at Orygen and will be kept for at least 25 years, but may be kept indefinitely.

7. I have been informed that the confidentiality of the information I provide will be safeguarded subject to any legal requirements; my data will be password protected and accessible only by the named researchers.

8. I understand that after I sign and return this consent form, it will be retained by the researcher.

Name of Participant (please print) ______________________________
Signature ____________________ Date ____________________

OPTIONAL CONSENT (please tick)

☐ I agree to my de-identified information being used for future research

☐ I do not agree to my de-identified information being used for future research

Initials of Participant ______________________________
Signature ____________________ Date ____________________

Signature of Researcher

Name of Researcher (please print) ______________________________
Signature ____________________ Date ____________________

Note: All parties signing the consent section must date their own signature.

PARENT/GUARDIAN TO SIGN

Third Party Consent Form

Centre of Youth Mental Health, University of Melbourne & Orygen

THE UNIVERSITY OF MELBOURNE

**Dated:**	09/06/2020
**Site:**	Orygen
**Full project title:**	Multimodal Approach to Preventing Suicide in Schools (MAPSS): A regionally-based trial of safeTALK and Reframe IT among secondary school students
**Project phase:**	2 (evaluation of Reframe IT)
**Responsible researcher:**	Associate Professor Jo Robinson<br>*Email:* jo.robinson@orygen.org.au
**Associate researchers:**	Professor Jane Pirkis, Professor Cathy Mihalopoulos, A/Professor Matthew Spittal, Dr Simon Rice, A/Professor Sarah Hetrick, Matthew Hamilton, Hok Pan Yuen, Michelle Lamblin, Sadhbh Byrne, Eleanor Bailey, Meaghan Dickens, Alexandra Boland, Karolina Krysinska, Alison Clarke, Louise La Sala, India Bellairs-Walsh, Caitlin Bleeker, Nicole Hill, Katrina Witt, Pinar Thorn, Zoe Teh.
**Funding sources:**	The National Health and Medical Research Council

1. I give my permission for ______________________________ to participate in this project and I will be provided with a written plain language statement to keep.
2. I understand that the purpose of this research is to investigate the impact of the Reframe IT website.
3. I understand that my child's participation in this project is for research purposes only.
4. I acknowledge that the possible effects of participating in this research project have been explained to my satisfaction.
5. I understand that my child's participation is voluntary and that they are free to withdraw from this project anytime without explanation or prejudice and to withdraw any unprocessed data that they have provided.

6. I understand that the data from this research will be stored at Orygen and will be kept for at least 25 years, but may be kept indefinitely, with the exception of MBS and PBS data which will be destroyed after 15 years.

7. I have been informed that the confidentiality of the information my child provides will be safeguarded subject to any legal requirements; their data will be password protected and accessible only by the named researchers.

8. I understand that after I sign and return this consent form, it will be retained by the researcher.

<u>OPTIONAL CONSENT (please tick)</u>

☐ I agree to my child's de-identified information being used for future research

☐ I do not agree to my child's de-identified information being used for future research

<u>Signature of Parent or Guardian</u>

Name of participant (please print)	______________________________
Name of parent or guardian	______________________________
Relationship to participant	______________________________
Signature of parent or guardian	________________ Date ________

<u>Signature of Researcher</u>

Name of Researcher (please print)	______________________________
Signature ______________________	Date ______________________

Note: All parties signing the consent section must date their own signature.

<u>Signature of Witness (ONLY IF THE PARENT/GUARDIAN CANNOT READ THIS FORM THEMSELVES)</u>

Name of Witness (please print)	______________________________
Signature ______________________	Date ______________________

* Witness is required when the parent or guardian cannot read the document for themselves.
